# Management Challenges of a Pregnant Woman With Undiagnosed Nephrotic Syndrome: Experience From Saudi Arabia

**DOI:** 10.7759/cureus.89347

**Published:** 2025-08-04

**Authors:** Marwa E Fadailu, Al Shamrani Saad M, Nadia A Alghilan, Marwa Ali

**Affiliations:** 1 Department of Obstetrics and Gynecology, Ministry of National Guard Health Affairs, Riyadh, SAU; 2 Department of Perinatology, Ministry of National Guard Health Affairs, Riyadh, SAU; 3 Department of Obstetrics and Gynecology, King Saud Bin Abdulaziz University for Health Sciences, Ministry of National Guard Health Affairs, Riyadh, SAU

**Keywords:** general nephrology, hypertension, nephrotic syndrome, pregnancy, saudi arabia

## Abstract

This case report discusses the overall care of a female patient with nephrotic syndrome secondary to non-pre-eclampsia-related hypertension in pregnancy, emphasizing the challenges and multidisciplinary treatment needed for desired results. The case presented here involves a 32-year-old pregnant woman with a history of unexplained primary infertility who conceived through in vitro fertilization (IVF). At 26 weeks and three days of gestation, she presented with symptoms suggestive of nephrotic syndrome, including lower limb swelling, facial puffiness, oliguria, and dark-colored urine. In her history, she had gestational hypertension and was treated with methyldopa. The first presentation and later management of this patient demonstrate the complexities involved in the management of pregnancy complicated by nephrotic syndrome. As part of the diagnostic workup, this patient had a detailed clinical assessment together with laboratory and imaging investigations, which substantiated the diagnosis of nephrotic syndrome. Management of the patient was made complicated by poorly controlled hypertension, the presence of proteinuria, and active inflammation, necessitating combined care by a multidisciplinary team. The management plan, performed at King Abdulaziz Medical City in Riyadh, included close monitoring of maternal and fetal parameters, pharmacological intervention for hypertension and infection, and supportive care to address the symptoms of nephrotic syndrome. This case highlights the complex interplay between nephrotic syndrome and pregnancy, emphasizing the critical need for early diagnosis, multidisciplinary management, and individualized therapeutic strategies. The patient’s favorable outcome, achieved through timely intervention (including renal biopsy and emergency cesarean delivery), demonstrates that, even in high-risk scenarios, a coordinated approach can optimize both maternal and fetal outcomes.

## Introduction

Nephrotic syndrome, characterized by heavy proteinuria (>3.5 g/day), hypoalbuminemia, and edema, poses unique diagnostic and therapeutic challenges when it occurs during pregnancy [1,2]. While nephrotic syndrome can occur independently of pregnancy, its co-existence with gestation significantly increases maternal-fetal risks, including accelerated hypertension, thromboembolic events, and preterm delivery [3,4].

The management complexity is compounded by the need to balance maternal treatment efficacy with fetal safety, particularly when distinguishing primary glomerular disease from pregnancy-specific conditions such as preeclampsia [5,6]. Nephrotic syndrome in pregnancy shows glomerular abnormalities of pathology that may determine its course with severe complications, such as acute kidney injury, thromboembolic phenomena, and unfavorable effects on the fetus [7]. As such, the management of nephrotic syndrome in this situation is intricate. It is a question of maximizing the mother's health and the risk posed to the fetus [8].

In this patient, the diagnosis of nephrotic syndrome (rather than preeclampsia) was supported by (1) early second-trimester onset (26 weeks), (2) selective proteinuria with severe hypoalbuminemia (18 g/dL), (3) absence of classic preeclampsia features (normal platelets/LFTs), and (4) persistence of proteinuria postpartum, later confirmed by biopsy. The case report describes the damaging consequences of this disorder in pregnancy if diagnosed and treated belatedly. Additionally, in this case, there is a need for a collaborative multidisciplinary team that can manage the medical intricacies brought about by this disease well. In the management of the patient, multiple life-saving procedures, including emergency C-section, ICU admission, and renal biopsy, were performed, which managed to stabilize the conditions of both the mother and the baby.

## Case presentation

A 32-year-old woman, gravida 2, at 26 weeks and three days of gestation, with an in vitro fertilization (IVF) pregnancy for unexplained primary infertility, presented to the ER on January 25, 2024, at King Abdulaziz Medical City in Riyadh. She complained of lower limb swelling, facial puffiness, oliguria, and dark-colored urine for five weeks.

Her medical history was significant only for gestational hypertension (GHTN), for which she was administered 250 mg of methyldopa twice daily. Upon examination, she appeared weak, with generalized body swelling and a puffy face. Vital signs revealed that her temperature was 36.9 °C, blood pressure (BP) was 139/93 mmHg, mean arterial pressure (MAP) was 107 mmHg, respiratory rate (RR) was 19 cycles/min, heart rate (HR) was 91 beats/minute, and oxygen saturation (SpO_2_) was 100% in room air.

Extremity examination revealed pitting edema in both feet and hands. Obstetrical examination showed a symphysis-fundal height of 26 weeks, cardiotocography (CTG) was reactive with a fetal HR (FHR) of 138 beats/min, and no contractions were detected.

Laboratory results indicated hemoglobin (Hb) of 135 g/dL, hematocrit of 0.34 L/L, leukocytes of 4.71 x 10^9^/L (with neutrophils of 79.0% and lymphocytes of 7%), platelets of 321 x 10^9^/L, erythrocyte sedimentation rate (ESR) of 5 mm/hour, creatinine of 48 umol/L, blood urea nitrogen (BUN) of 4.0 mmol/L, albumin of 18 g/dL, uric acid of 254 μmol/L, potassium of 5.0 mmol/L, sodium of 111 mmol/L, calcium of 1.85 mmol/L, and magnesium of 0.62 mmol/L. Liver function tests revealed a total bilirubin level of 5.0 μmol/L, total protein level of 43.6 g/L, aspartate aminotransferase (AST) of 38 U/L, alanine aminotransferase (ALT) of 19 U/L, lactate dehydrogenase (LDH) of 373 U/L, and lactic acid of 1.63 mmol/L. The coagulation profile was within normal limits with an international normalized ratio (INR) of 0.83, prothrombin time (PT) of 9.70 seconds, and partial thromboplastin time (PTT) of 34.50 seconds. Urinalysis revealed proteinuria, hematuria, and various casts. Blood gas analysis showed normal pH, pCO_2_, pO_2_, HCO_3_, and base excess (BE). Blood serum osmolality was 228 mOsm/kg. More details about the laboratory tests are shown in Table 1, with the reference ranges per hospital protocol.

**Table 1:** Laboratory findings The reference ranges are as per the hospital protocol. AST: alanine transaminase; ALT: alanine aminotransferase; HCT: hematocrit; LDH: lactate dehydrogenase; PTT: partial thromboplastin time; PT: prothrombin time

Variables	Result	Reference range
Hb	135 g/dL	120-160 g/dL
HCT	0.34 L/L	0.36-0.54 L/L
Leukocyte	4.71	4-11 x 10^9^
Neutrophil	79%	2-7.5 x 10^9^
Lymphocytes	7%	1- 4.4 x 10^9^
Platelets	321 x 10^9^	150-400 x 10^9^
ESR	5 mm/hr	0-20 mm/hr
Creatinine	48	44-88 umol/L
Blood urea nitrogen (BUN)	4.0	2.5-6.7
Albumin	18	35-50 g/dL
Uric acid	254 umol/L	150-370 umol/L
Potassium	5.0 mmol/L	3.5-5.1 mmol/L
Sodium	111 mmol/L	136-145 mmol/L
Calcium	1.85 mmol/L	2.1-2.55 mmol/L
Magnesium	0.62 mmol/L	0.66-1.07 mmol/L
Total bilirubin	5 umol/L	~ 20.5 umol/L
Total protein level	43.6	-
AST	38 U/L	~ 34 U/L
ALT	19 U/L	11-34 U/L
LDH	373 U/L	125-220 U/L
PTT	34.50 seconds	24.84-32.96 seconds
PT	9.70 seconds	9.38-12.34 seconds
INR	0.83	0.8-1.2
Blood serum osmolality	228 mOsm/kg	
Lactic acid	1.63 mmol/L	

The patient was admitted to the ICU for electrolyte correction. Further laboratory workup showed proteinuria, and fetal ultrasonography (USG) revealed an average fetal size of 25 weeks with normal Doppler velocimetry. Dexamethasone was initiated for fetal lung maturation, and magnesium sulfate (MgSO_4_) was administered for neuroprotection. Despite initial stabilization, the patient's BP became uncontrolled, reaching 156/100 mmHg.

A multidisciplinary team (MDT) meeting was held involving the obstetrician, neonatal intensive care unit (NICU), maternal-fetal medicine (MFM), ICU, cardiology, nephrology, rheumatology, internal medicine (IM), and anesthesia. On February 6, 2024, the patient underwent an emergency lower-segment cesarean section (LSCS) because of reversed end-diastolic flow in the umbilical artery (UAD). Intraoperative findings included high vascularity, abdominal ascetic fluid, and severe edema with fluid in the subcutaneous layer. Postoperatively, the patient developed shortness of breath (SOB) and fever on day three. Computed tomography (CT) pulmonary angiography revealed no radiographic evidence of pulmonary embolism (PE). A sepsis workup was initiated with empirical antibiotics (meropenem and vancomycin). Blood and wound cultures isolated *Klebsiella pneumoniae*, which was sensitive to meropenem but resistant to vancomycin. The antibiotic regimen was de-escalated to intravenous meropenem monotherapy (1 g every eight hours), with discontinuation of vancomycin. Therapeutic drug monitoring ensured optimal dosing, and renal function was closely monitored given the patient’s acute kidney injury (AKI). By day five, the patient’s BP was stabilized with oral labetalol (100 mg twice daily), extended-release nifedipine (30 mg once daily), and intravenous furosemide (40 mg twice daily) to manage fluid overload.

CT of the abdomen demonstrated postoperative changes consistent with recent cesarean delivery, including a small subcutaneous hematoma (measuring 2.5 × 1.8 cm) in the anterior abdominal wall and diffuse anasarca with significant subcutaneous edema, peritoneal fluid, and bowel wall thickening. No evidence of abscess formation, active bleeding, or intra-abdominal collections was observed. On the sixth hospital day, the patient developed recurrent febrile episodes (peaking at 39.2°C), persistent tachycardia (HR: 120-140 bpm), and worsening SOB. These findings prompted immediate evaluation for potential sepsis progression or thromboembolic complications. Chest radiography showed interstitial pulmonary edema, and lower-limb Doppler showed massive lower-limb edema without deep vein thrombosis (DVT). The infectious disease (ID) team continued meropenem, stopped vancomycin, and started linezolid.

**Figure 1:**
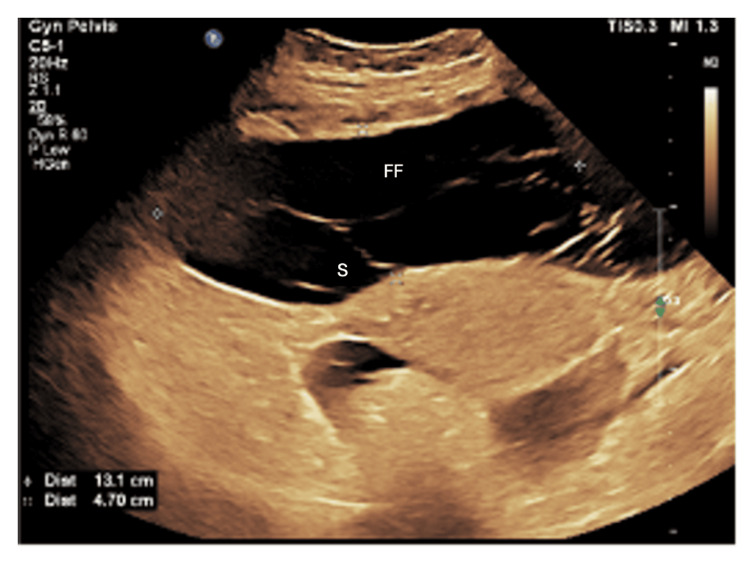
Free fluid with multiple internal sepatations inside detected by ultrasonopgraphy This is an original image. FF: Free fluid, S: Separation

The patient received a packed red blood cell (PRBC) unit for an Hb of 7.8 g/dL. By day 10, the patient had continued to experience SOB, fever, and tachypnea. A CT revealed worsening ascites, and retrograde cystography showed no bladder injury. After reviewing the patient's ongoing febrile status and culture results, the ID team made the decision to transition antimicrobial therapy from intravenous linezolid to intravenous tigecycline for broader-spectrum coverage. On day 11, the patient's condition deteriorated, with increased HR, RR, and continuous fever. Continuous positive airway pressure (CPAP) was initiated, and abdominal fluid aspiration revealed clear serous fluid. On hospital day 12, the patient continued intravenous tigecycline (50 mg every 12 hours after a 100 mg loading dose) for persistent wound infection. The surgical team implemented twice-daily wound care with silver alginate dressings to address the contaminated abdominal incision, along with strict infection control measures. A CT urogram was performed to rule out ureteric injury. By day 15, the nephrology team diagnosed AKI secondary to hypoperfusion (likely due to hypovolemia or sepsis) and initiated daily intravenous 20% albumin (100 mL/day for five days) to restore intravascular volume and improve renal perfusion, alongside close monitoring of fluid balance and renal function. Metoprolol (37.5 mg) was administered twice daily. On day 17, the patient showed dramatic improvements in RR and HR. On day 20, the patient underwent repeat abdominal/pelvic CT amid clinical instability (HR: 131 bpm, RR: 42/min, T: 39°C, BP: 131/88 mmHg) to assess for progressive infection or thrombosis. The urine output was 400 mL in four hours. CT revealed extensive fluid collections, likely representing the infected flu, for which a transabdominal drainage tube was inserted, confirming the ultrasound findings of the moderate to severe free fluid with multiple final internal separations inside (Figure 1). On day 21, an MDT meeting was held, and the nephrology team decided to proceed with a kidney biopsy due to massive proteinuria with normal kidney function tests.

Following renal biopsy confirmation of nephrotic syndrome, the patient was maintained on lisinopril 10 mg daily and nifedipine 120 mg daily at discharge. Three-month follow-up demonstrated partial remission (proteinuria: <2 g/day) with stable renal function. Long-term management focuses on monitoring for disease relapse, hypertension control, and chronic kidney disease progression, with particular attention to the 30-40% recurrence risk in future pregnancies, necessitating specialized pre-conception counseling.

## Discussion

Pregnancy in women suffering from nephrotic syndrome has hitherto been considered to be a challenging and complex task, as a multidisciplinary approach is often needed [8]. In the case of this patient, it is illustrative of how necessary it is to find the right balance in managing the mother while considering the developing fetus. The preterm neonate was admitted to the NICU for respiratory support and management of complications of extreme prematurity. The baby survived the neonatal period and was discharged in stable condition with ongoing follow-up for prematurity-related monitoring. The history was remarkable for evidence of lower limb swelling, facial puffiness, reduction in urinary output, and dark-colored urine. A thorough diagnostic workup was done, and prompt action was taken.

Nephrotic syndrome pathophysiology is anchored in glomerular injury that manifests in excess proteinuria, low levels of albumin in blood, and swollen tissues. These changes can cause grave consequences in both the mother and fetus, such as acute renal failure, thromboembolic diseases, and poor fetal outcomes [9,10]. In this patient's case, however, uncontrolled high blood pressure and signs of infection made them seek the help of many specialists, including obstetricians, nephrologists, infectious disease specialists, and the intensive care unit.

Initial management included close monitoring of maternal and fetal parameters, pharmacological intervention for hypertension and infection, and supportive care to address the symptoms of nephrotic syndrome. Antihypertensive agents such as labetalol, nifedipine, and diuretics such as furosemide helped control the patient's blood pressure. The administration of albumin and metoprolol greatly benefited in managing the fluid and electrolyte loss associated with nephrotic syndrome [11,12].

It was essential to decide on an emergency cesarean section, as this facilitated the birth of the fetus, and, at the same time, the risks of the mother's condition worsened. The emergency LSCS, while necessitating extreme preterm delivery, was ultimately life-saving for both mother and fetus. This decision exemplifies the critical balance in obstetric nephrology, when maternal organ dysfunction and placental insufficiency coincide, delivery remains the definitive intervention despite its associated risks.

The post-operative course was complicated by sepsis, requiring aggressive antibiotic therapy and intensive care support. Meropenem and vancomycin, followed by linezolid and tigecycline, reflect the importance of tailored antimicrobial treatment based on culture results in managing infections in this setting [13,14].

The renal biopsy was also critical in establishing a diagnosis of the nephrotic syndrome, as it would guide the patient's long-term care. The biopsy also contributed to the etiology distinguishing nephrotic syndrome, thus aiding in formulating placement options. Biopsy results directed placement into steroid therapy or calcineurin inhibitors, with concurrent angiotensin-converting enzyme (ACE) inhibitors for renoprotection [15,16].

This case's extensive laboratory and imaging findings demonstrate the complex pathophysiology of pregnancy-related nephrotic syndrome, which causes severe proteinuria (>3.5 g/day) and significant hypoalbuminemia (18 g/dL) due to glomerular barrier disruption. These biochemical changes enhanced maternal edema and thrombosis risks and fetal development issues, such as intrauterine growth restriction. The renal biopsy differentiated intrinsic glomerular disease from preeclampsia, enabling targeted therapy such as immunosuppression for podocyte injury and anticoagulation for hypoalbuminemia-induced hypercoagulability. The patient's favorable response to corticosteroids and ACE inhibitors postpartum supports this mechanism-based therapeutic strategy, emphasizing the necessity of precise diagnosis and personalized management in these complex cases to optimize outcomes for both mother and fetus. This approach aligns with current evidence demonstrating that (1) early immunosuppression improves proteinuria in pregnancy-associated nephrotic syndrome [17] and (2) ACE inhibitors postpartum reduce progression to chronic kidney disease [18].

In this situation, nephrotic syndrome has to be detected and treated as early as possible during pregnancy, reflecting the role of a multidisciplinary team in managing such cases. Engaging specialists through various specialties made attending to all client concerns possible, benefiting both the mother and child. This case report highlights the approach used in the management of nephrotic syndrome in pregnancy, which is a challenging clinical problem and requires a multi-dimensional approach.

## Conclusions

This case highlights the complex interplay between nephrotic syndrome and pregnancy, emphasizing the critical need for early diagnosis, multidisciplinary management, and individualized therapeutic strategies. The patient’s favorable outcome, achieved through timely intervention (including renal biopsy and emergency cesarean delivery) that demonstrates that even in high-risk scenarios, a coordinated approach can optimize both maternal and fetal outcomes.
